# Development and Immune Efficacy Evaluation of Two Live Triple-Gene-Deleted Vaccine Candidates Against Bovine Herpesvirus Type 1

**DOI:** 10.3390/ani16111606

**Published:** 2026-05-25

**Authors:** Yiping Gu, Hongzuo Duan, Congyun Ji, Hanyu Lin, Yuxin Lai, Jianwei Zhang, Cun Zhang, Suxin Huo, Zheng Ni, Tao Yun, Weicheng Ye, Jionggang Hua, Liu Chen, Yuan Fu, Yinchu Zhu, Zihao Pan

**Affiliations:** 1 College of Veterinary Medicine, Nanjing Agricultural University, Nanjing 210095, China; 2 Zhejiang Key Laboratory of Livestock and Poultry Biotech Breeding, Key Laboratory of Livestock and Poultry Resources (Poultry) Evaluation and Utilization, Institute of Animal Husbandry and Veterinary Sciences, Zhejiang Academy of Agricultural Sciences, Hangzhou 310021, China; 3 MOE Joint International Research Laboratory of Animal Health and Food Safety, Nanjing 210095, China

**Keywords:** infectious bovine rhinotracheitis, bovine herpesvirus type 1, triple-gene deletion, live vaccine, immunogenicity evaluation

## Abstract

Bovine herpesvirus type 1 (BoHV-1) causes severe respiratory and reproductive diseases in cattle, leading to significant economic losses worldwide. Current inactivated vaccines in China fail to induce robust cellular immunity and cannot differentiate infected from vaccinated animals (DIVA). To address these limitations, we constructed two triple-gene-deleted BoHV-1 vaccine candidates, ∆T3 (∆*TK*-∆*gE/gI*) and ∆g3 (∆gG-∆gE/gI), using Red/ET two-step recombination technology based on an infectious BAC clone. Their safety and immunogenicity were evaluated in a rabbit model. Both deletion mutants exhibited growth kinetics similar to the wild-type strain but with significantly reduced viral titers and plaque areas. The triple-gene deletion mutants showed superior safety compared with single-gene deletion mutants, with minimal clinical signs and low viral shedding. Immunization with ∆T3 and ∆g3 induced robust *gB*-specific and neutralizing antibody responses. Following challenge with the virulent BoHV-1 BC01 strain, vaccinated rabbits maintained normal body temperatures, showed significantly reduced viral shedding (approximately 100-fold), and exhibited milder pulmonary lesions. These findings demonstrate that ∆T3 high-dose and ∆g3 low-dose regimens are promising DIVA-compatible vaccine candidates, offering an effective strategy for IBR control and eradication programs.

## Simple Summary

Bovine herpesvirus type 1 (BoHV-1) causes severe respiratory disease and reproductive disorders in cattle, leading to major economic losses worldwide. Current vaccines in China have limitations, as they cannot distinguish vaccinated animals from infected ones. In this study, we developed two new live vaccines by removing three viral genes (rBoHV-∆gG-∆gE/gI and rBoHV-∆TK-∆gE/gI). We tested their safety and effectiveness in rabbits. The results show that both vaccines were safe and induced strong immune responses. After exposure to the virus, vaccinated rabbits showed no fever, significantly reduced virus shedding, and less lung damage. Notably, the rBoHV-∆TK-∆gE/gI vaccine worked best at a high dose, while the rBoHV-∆gG-∆gE/gI vaccine was effective even at a low dose. These findings suggest that our new vaccines could help control BoHV-1 infections and support eradication programs by allowing differentiation between vaccinated and naturally infected animals.

## 1. Introduction

Infectious bovine rhinotracheitis (IBR), caused by *Bovine Herpesvirus type 1* (BoHV-1), is an acute, contagious disease associated with severe respiratory manifestations, immunosuppression, and lifelong latent infection in cattle [1,2]. The global economic burden of BoHV-1 infection exceeds $3 billion annually, primarily due to reduced productivity, trade restrictions, and control costs [3]. Once established, latent infection allows the virus to persist in sensory ganglia, with periodic reactivation and shedding following stress or immunosuppression, perpetuating transmission cycles within herds and complicating eradication programs [4].

BoHV-1 is a member of the family Herpesviridae, subfamily Alphaherpesvirinae, with a double-stranded DNA genome of approximately 135–140 kb encoding around 70 proteins [5,6,7]. Among these, glycoproteins such as *gB*, *gC*, and *gD* are major immunogens, while others like *gG*, *gE/gI*, and *TK* contribute to viral pathogenesis, immune evasion, and latency [8,9]. The *gG* protein has been implicated in immunosuppression via chemokine binding, thereby modulating the host inflammatory response and facilitating viral dissemination [10,11]. The *gE/gI* complex facilitates cell-to-cell spread and inhibits interferon signaling [12,13,14]. Thymidine kinase (*TK*) is essential for neurovirulence and reactivation from latency [15]. Collectively, these virulence-associated genes represent promising targets for genetic attenuation and marker vaccine development.

Current vaccination strategies in China rely primarily on inactivated vaccines. However, these vaccines fail to induce robust cellular immunity and do not allow serological differentiation between infected and vaccinated animals (DIVA). Their inability to elicit cytotoxic T-cell responses limits their efficacy in clearing virus-infected cells, while the lack of DIVA compatibility hampers serosurveillance efforts essential for eradication. In contrast, gene-deleted marker vaccines enable DIVA implementation and have been instrumental in IBR eradication programs in several European countries [16]. Such vaccines, typically based on deletions of immunomodulatory genes, allow for the distinction between vaccinated and naturally infected animals through companion diagnostic tests, thereby facilitating herd-level monitoring and gradual disease elimination.

To address the limitations of existing vaccines, we constructed two triple-gene-deleted BoHV-1 mutants, ∆*TK-∆gE/gI* (∆T3) and ∆*gG-∆gE/gI* (∆g3), and evaluated their safety, immunogenicity, and protective efficacy in a rabbit model. This study provides a foundation for developing next-generation, DIVA-compatible BoHV-1 marker vaccines suitable for eradication strategies. By systematically deleting virulence-associated genes while retaining major immunogenic glycoproteins, these candidates are designed to balance attenuation with immunogenicity, offering the potential for safe and effective control of IBR in endemic regions.

## 2. Materials and Methods

### 2.1. Cells and Viruses

A virulent BoHV-1 strain, isolated in our laboratory from clinical samples, was designated as BoHV-1 BC01 (GenBank: PV550781.1). MDBK cells were purchased from the American Type Culture Collection (ATCC). The pBoHV-1-BAC infectious clone, the GS1783 *E. coli* strain, the pCAGGS-NLS/Cre plasmid, and the pLAY2KAN plasmid carrying a kanamycin resistance gene and an I-SceI site were provided by the Institute of Animal Husbandry and Veterinary Medicine, Zhejiang Academy of Agricultural Sciences. All primers used in this study are listed in Table S1.

### 2.2. Reagents and Consumables

The experimental reagents and consumables used in this study are shown in Table 1 and Table 2, respectively.

### 2.3. Development and Identification of Recombinant Transfer Plasmids

**Figure 1:**
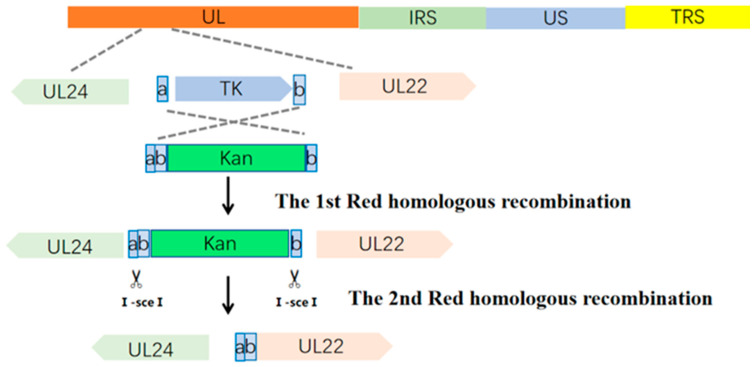
Schematic diagram of the two-step recombination for BoHV-1 BAC-ΔTK.

The triple-gene deletion recombinant plasmids were constructed using the “two-step Red/ET recombination” technique. Using the *TK* gene as an example, the knockout strategy is illustrated schematically in Figure 1. Targeting fragments for the three genes (*TK*, *gE/gI*, and *gG*) were individually amplified by polymerase chain reaction (PCR) from the pLAY2KAN plasmid using 2 × TOROBlue Flash KOD Dye Mix (Toroivd, Shanghai, China). After gel extraction, the targeting fragments were electroporated into prepared competent pBoHV-1-BAC GS1783 cells (1.8 kV, 200 Ω, 25 μF). Following a second round of homologous recombination screening, the three single-gene deletion mutants were isolated. The pBoHV-1-BAC plasmid was extracted via alkaline lysis. Using the same method, targeting fragments for TK and gG were electroporated into prepared competent pBoHV-1-Δ*gE/gI* GS1783 cells. After two-step homologous recombination, two recombinant plasmids, pBoHV-1-Δ*TK*-Δ*gE/gI* and pBoHV-1-Δ*gG*-Δ*gE*/*gI*, were obtained. Both recombinant plasmids were verified by PCR using gene-specific primers designed for the three target genes. Upon confirmation of correct construction, the plasmids were stored at −20 °C for subsequent use.

### 2.4. Rescue of the Recombinant Viruses

Following the calcium phosphate transfection method [17], the plasmids pBoHV-1-∆*gG*, pBoHV-1-∆*TK*, pBoHV-1-∆*gE/gI*, pBoHV-1-∆*gG*-∆*gE/gI*, and pBoHV-1-∆*TK*-∆*gE/gI* were transfected into MDBK cells. The viruses were harvested when fluorescence was observed and fluorescent plaques reached approximately 90% confluence, yielding the corresponding recombinant viruses: rBoHV-1-∆*gG*, rBoHV-1-∆*TK*, rBoHV-1-∆*gE/gI*, rBoHV-1-∆*gG*-∆*gE/gI*, and rBoHV-1-∆*TK*-∆*gE/gI*. Among these, rBoHV-1-∆*gG*-∆*gE/gI* and rBoHV-1-∆*TK*-∆*gE/gI* were designated as ∆g3 and ∆T3, respectively.

### 2.5. Growth Curves of the Recombinant Viruses

MDBK cells were seeded into 6-well plates. The following day, the cells were infected with either ∆g3 or ∆T3 at a multiplicity of infection (MOI) of 0.1. After 2 h of adsorption, the inoculum was replaced with DMEM supplemented with 2% fetal bovine serum (FBS). The infections were set up in triplicate wells, and the plates were incubated at 37 °C in a 5% CO_2_ incubator. Viral supernatants were collected at 6, 12, 24, 36, 48, and 72 h post-infection (hpi), aliquoted, and stored at −80 °C. The viral samples were subjected to three freeze-thaw cycles before being titrated on 96-well plates seeded with MDBK cells. Cytopathic effect (CPE) was observed 2 days post-infection, and the viral titer (TCID_50_/mL) was calculated using the Reed–Muench method. Growth curves were subsequently plotted, and the growth kinetics of the two recombinant viruses were analyzed in comparison with their parental strain. All experiments were independently performed in triplicate.

### 2.6. Plaque Area of Recombinant Viruses

Frozen stocks of the ΔT3, Δg3, and wild-type viruses were serially diluted and inoculated onto monolayers of MDBK cells in 6-well plates. After 2 h of adsorption, the inoculum was replaced with an overlay of DMEM containing 1% methylcellulose. The plates were then incubated at 37 °C in a 5% CO_2_ incubator for 96 h. Following incubation, the overlay was removed, and the cell monolayers were fixed overnight with 4% paraformaldehyde. The next day, the fixed monolayers were stained with 1% crystal violet for approximately 10 min. After rinsing with water to remove excess stain, distinct plaques became clearly visible to the naked eye. Subsequently, 100 random plaques per group were imaged under a microscope. The plaque areas were measured using ImageJ (version Image J2) software, and the data from the three virus groups were analyzed using GraphPad Prism software (version 9.4).

### 2.7. Animal Experiments

#### 2.7.1. Safety Evaluation

Fifty New Zealand White rabbits (purchased from SPF-Biotech, Hangzhou, China) were randomly divided into 10 groups. The three single-gene deletion mutant groups were each inoculated with 1.0 × 10^6^ TCID_50_/mL. The two triple-gene deletion mutant groups were further divided into high-dose (1.0 × 10^7^ TCID_50_/mL) and low-dose (1.0 × 10^6^ TCID_50_/mL) subgroups. A wild-type virus group (1.0 × 10^6^ TCID_50_/mL) and a blank control group (1 mL DMEM) were included as controls. The detailed grouping information is shown in Table 3. Following injection, clinical manifestations, body temperature fluctuations, and viral shedding in rabbits were monitored to compare the outcomes between the gene-deleted virus groups and the wild-type virus group.

#### 2.7.2. Immunogenicity Evaluation

The two triple-gene deletion mutant groups were further divided into high-dose (1.0 × 10^7^ TCID_50_/mL) and low-dose (1.0 × 10^6^ TCID_50_/mL) subgroups. The inactivated vaccine (purchased from Sinovet Co., Ltd., Taizhou, China) group was immunized according to the manufacturer’s instructions. A booster immunization of inactivated vaccine was administered on day 21. All immunizations were given via intramuscular injection in the hind leg. At day 28 post-immunization, the inactivated vaccine group, the two triple-gene deletion virus groups, and the DMEM control group were challenged via intranasal inoculation with 1.0 × 10^8^ TCID_50_/mL of the BoHV-1 BC01 strain. Detailed information on the challenge route and dose is shown in Table 4.

### 2.8. Clinical Signs and Viral Shedding Detection

Following challenge, the rectal temperature of rabbits was measured daily during the first week, and clinical signs, including mental state, appetite, respiratory symptoms, and ocular discharge, were observed. Nasal swabs were collected daily for the first week post-inoculation and then every other day until viral detection became negative. The swabs were vortexed thoroughly in tubes containing 500 uL of sterile PBS. Viral nucleic acids were extracted from the nasal swabs using a Magnetic Bead-based Animal Virus DNA/RNA Rapid Extraction Kit (purchased from Xingchun Biotechnology Co., Ltd., Changzhou, China). Viral loads in swabs and tissues were quantified by a TaqMan qPCR method targeting the BoHV-1 UL1 region (Primers/Probe in Table S1), which was previously established in our laboratory.

At least 2 mL of blood was collected weekly from each rabbit. The samples were kept at 4 °C overnight to allow for serum separation, followed by centrifugation at 1000× *g* for 10 min. The resulting serum was aliquoted and stored at −80 °C for subsequent detection of specific antibodies and neutralizing antibodies.

Lung samples were collected from each rabbit at the third week post-challenge. At necropsy, the rabbits were euthanized by injecting an air bolus into the marginal ear vein. The lungs were carefully excised en bloc, rinsed gently with ice-cold sterile PBS to remove residual blood, and photographed using a digital camera(Nikon Corporation (Tokyo, Japan) under consistent lighting conditions. Macroscopic lung lesions (consolidation, hemorrhage, and necrosis) were evaluated by two independent observers who were blinded to the group allocation. The percentage of the lung surface area affected by gross lesions was scored using a semi-quantitative system: 0 = no visible lesions; 1 = 1–25% involvement; 2 = 26–50%; 3 = 51–75%; 4 = 76–100%. The scores from the two observers were averaged for each animal, and group means were calculated for comparison. The tissue specimens were fixed in 4% paraformaldehyde for 48 h and then embedded in paraffin. The sections were stained with hematoxylin and eosin (H&E) for histopathological examination.

### 2.9. Detection of Serum Antibodies

The gB and gE antibody blocking rate and the gB antibody titer at day 28 were detected using a commercial blocking ELISA kit (purchased from Ingenasa, Madrid, Spain). The gE antibodies were detected to differentiate between animals inoculated with the gene-deleted strains and those vaccinated with inactivated vaccine or infected with wild-type virus.

### 2.10. Neutralization Assay

Serum samples were inactivated by heating at 56 °C for 30 min, then subjected to serial dilution in 96-well cell culture plates. For each dilution, four replicates per sample were prepared. The diluted serum samples were incubated with 100 TCID_50_ of BoHV-1 BC01 virus at 37 °C in a 5% CO_2_ incubator for 1 h. The serum–virus mixtures were then transferred onto 96-well plates containing MDBK cells and cultured for 2 days at 37 °C in a 5% CO_2_ incubator. The neutralizing antibody titer, defined as the highest serum dilution that inhibited BoHV-1 infection, was calculated using the Reed–Muench method.

### 2.11. Statistical Analysis

The data are organized and are presented as mean ± standard deviation (SD). Differences between groups were determined by *t*-test and one-way ANOVA using GraphPad Prism software (version 10.6). A *p*-value of less than 0.05 was considered statistically significant (* *p* < 0.05; ** *p* < 0.01; *** *p* < 0.001).

## 3. Results

### 3.1. Identification of the Recombinant Viruses

**Figure 2:**
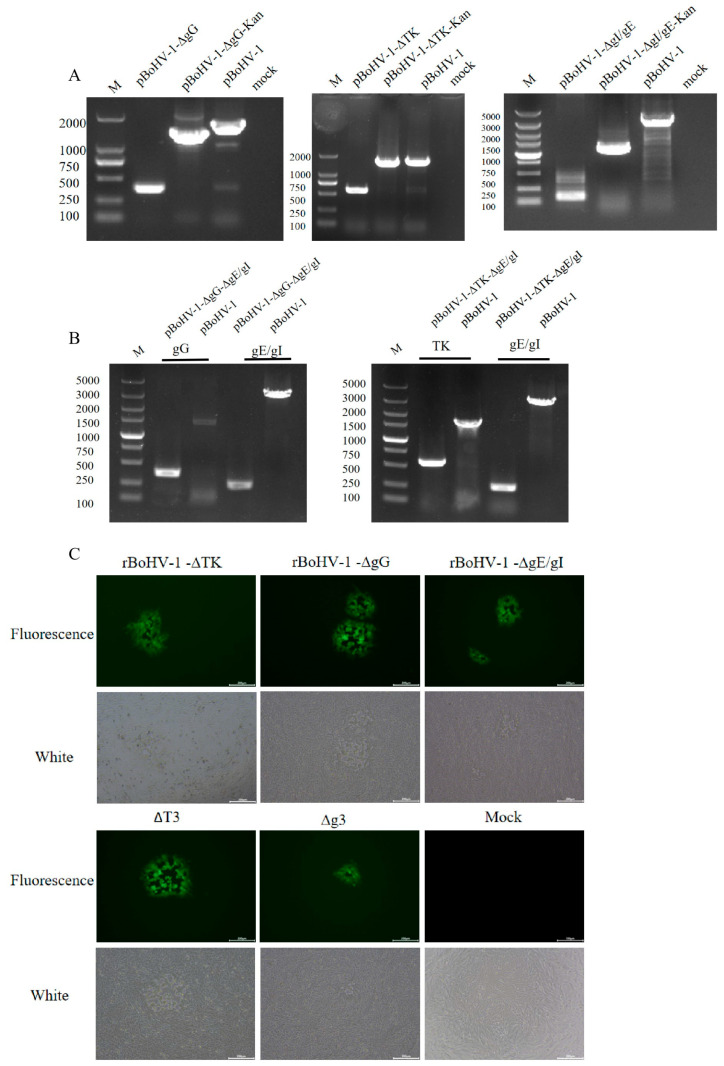
Construction and identification of recombinant BoHV-1. (**A**) PCR verification of the three single-gene deletion recombinant plasmids. Using specific primers for the TK, gG, and gE/gI genes. (**B**) PCR identification of the two triple-gene deletion recombinant plasmids. (**Left panel**): PCR verification of p∆g3; (**right panel**): PCR verification of p∆T3. (**C**) Observation of five recombinant plasmids in MDBK cells at 48 h post-transfection using the calcium phosphate method. The presence of green fluorescence indicates the successful rescue of this virus.

The two triple-gene deletion recombinant plasmids, the three single-gene deletion recombinant plasmids, and the three single-gene first-step homologous recombination products were extracted and used as templates for PCR amplification with primers Δ*TK*-JD-F, Δ*TK*-JD-R, Δ*gG*-JD-F, Δ*gG*-JD-R, Δ*gI/gE*-JD-F, and Δ*gI/gE*-JD-R. The results confirm that the three single-gene deletion mutants were successfully generated as expected (Figure 2A). The two triple-gene deletion mutants were also confirmed as expected (Figure 2B). Subsequently, the five recombinant plasmids were transfected into MDBK cells using the calcium phosphate transfection method. At 48 h post-transfection, green fluorescent plaques were observed under a fluorescence microscope (Figure 2C), indicating that the gene-deleted viruses were successfully rescued.

### 3.2. Growth Kinetics and Plaque Area of the Recombinant Viruses

**Figure 3:**
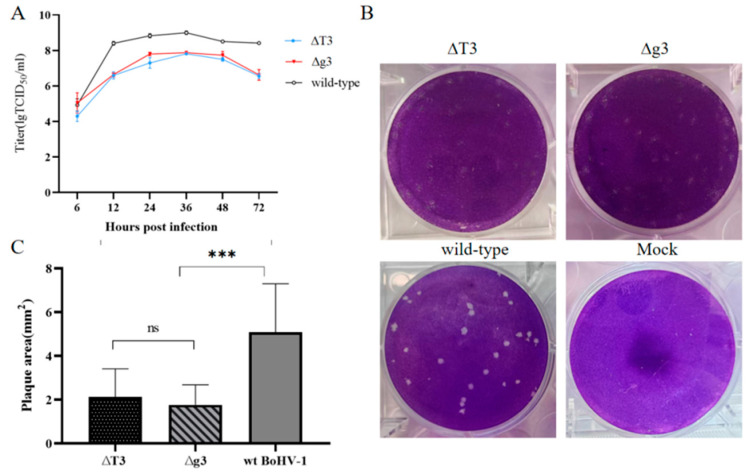
Growth characteristics of ∆g3 and ∆T3. (**A**) Viral growth curve. MDBK cells were infected at MOI = 0.1; supernatants were collected at indicated time points and titers expressed as TCID50/mL, calculated by the Reed–Muench method. (**B**) MDBK monolayers were overlaid with 1% low-melting agarose post-infection and stained to visualize plaques. (**C**) Plaque areas of ∆T3, ∆g3, and the wild-type strain. Significance levels are indicated as *** *p* < 0.001.

In this study, the one-step growth curves of these recombinant viruses (rBoHV-1-∆g3, rBoHV-1-∆T3) and the wild-type strain were determined and compared in MDBK cells. The results demonstrate that all virus strains exhibited similar replication kinetics, with viral titers peaking at 36 h post-infection. The peak titers at 36 h were 1.0 × 10^7.88^ TCID_50_/mL for rBoHV-1-∆g3, 1.0 × 10^7.70^ TCID_50_/mL for rBoHV-1-∆T3, and 1.0 × 10^8.98^ TCID_50_/mL for the wild-type strain (Figure 3A). The results reveal no significant differences in viral titers between the two triple-gene deletion viruses (rBoHV-1-∆g3 and rBoHV-1-∆T3) at any time point, indicating comparable in vitro replication capacities. Notably, the titers of all recombinant viruses were significantly lower than that of the wild-type strain (*p* < 0.001).

rBoHV-1-∆g3, rBoHV-1-∆T3, and the wild-type strain were used to infect MDBK cells. At 96 h post-infection, the cells were fixed overnight and stained, after which 100 viral plaques were photographed for each virus. As shown in Figure 3B, the plaque areas formed by the two recombinant viruses were visually apparent and significantly smaller than those of the wild-type strain. The area of each plaque was measured using ImageJ software, and the mean plaque area was calculated. The mean plaque areas of rBoHV-1-∆g3, rBoHV-1-∆T3, and the wild-type strain were 1.77 mm^2^, 2.13 mm^2^, and 5.08 mm^2^, respectively (Figure 3C). The plaque areas of both deletion mutants were significantly smaller than those of the parental wild-type strain (*p* < 0.001).

### 3.3. Safety Evaluation

**Figure 4:**
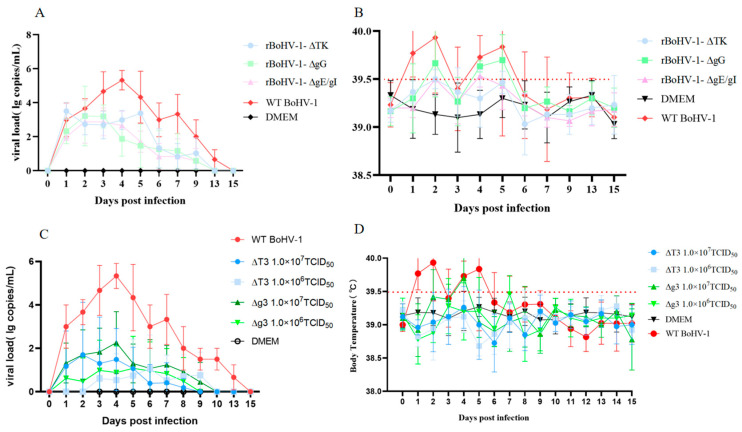
Post-immunization rectal temperature and viral load in nasal swabs from rabbits. (**A**) Viral load in nasal swabs from rabbits immunized with single-gene deletion mutants. (**B**) Rectal temperature changes in rabbits immunized with single-gene deletion mutants, the red dotted line indicates the upper limit of normal rectal temperature in rabbits. (**C**) Viral load in nasal swabs from rabbits immunized with triple-gene deletion mutants. (**D**) Rectal temperature changes in rabbits immunized with triple-gene deletion mutants, the red dotted line indicates the upper limit of normal rectal temperature in rabbits.

The safety profiles of three single-gene deletion recombinant viruses (rBoHV-1-∆*gG*, rBoHV-1-∆*gE/gI*, and rBoHV-1-∆*TK*) and two triple-gene deletion recombinant viruses (∆T3 and ∆g3) were evaluated in rabbits and found to exhibit marked differences. Regarding clinical observations (Table 5), immunization with the three single-gene deletion mutants induced adverse reactions of varying severity. In the rBoHV-1-∆*gG* group, some rabbits developed clinical signs including nasal discharge and increased ocular secretions, accompanied by notable body temperature fluctuations (Figure 4B). Temperatures exceeded the normal range by day 2 post-immunization and rose again to febrile levels on days 4–5. Notably, the two triple-gene deletion mutant groups exhibited markedly milder clinical symptoms. In the ∆T3 group, all rabbits remained completely asymptomatic. In the ∆g3 group, clinical signs were mild and self-limiting; one rabbit in the low-dose cohort developed transient sneezing and nasal discharge that resolved within three days, and only two rabbits in the high-dose cohort displayed symptoms. This favorable safety profile was further supported by body temperature monitoring, which showed that febrile responses were attenuated and shorter in duration in these groups (Figure 4D). In terms of viral shedding, the three single-gene deletion mutants exhibited a shedding period of approximately nine days post-immunization, with peak viral titers reaching 10^3.2^ to 10^3.5^ copies/mL (Figure 4A). The levels of viral shedding were reduced compared with the wild-type strain; a notable residual risk of viral transmission persisted. Indeed, the viral shedding was substantially attenuated in the triple-gene deletion mutant groups (Figure 4C). In the low-dose ∆T3 and ∆g3 groups, viral shedding was detected in only two rabbits per group, with peak titers of approximately 10^1.06^ and 10^1.08^ copies/mL, respectively. In the high-dose groups, shedding was observed in three rabbits for ∆T3 (peak titer about 10^1.7^ copies/mL) and four rabbits for ∆g3, which remained markedly lower than those induced by the single-gene deletion mutants. Above all, the triple-gene deletion mutants exhibited significantly better performance than the single-gene deletion mutants in terms of body temperature stability, control of clinical symptoms, and viral shedding capacity, highlighting the additive attenuating effect of multi-gene deletion and demonstrating a higher safety profile for vaccine development.

### 3.4. Immunogenicity Analysis of the Two Triple-Gene Deletion Mutants

#### 3.4.1. BoHV-1 Neutralizing Antibody Response

**Figure 5:**
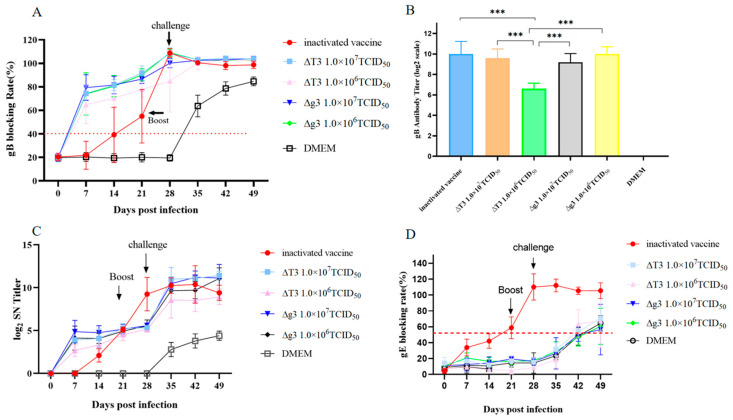
Humoral immune responses post-immunization. (**A**) BoHV-1 gB antibody blocking rate, (**B**) gB antibody titers at day 28, significance levels are indicated as *** *p* < 0.001, (**C**) neutralizing antibody titers were determined, and (**D**) gE antibody blocking rate. The red line indicates the negative-positive threshold for gB and gE antibodies.

As shown in Figure 5C, neutralizing antibody levels in all attenuated vaccine groups gradually increased between days 7 and 14 post-immunization. In contrast, no neutralizing antibodies were detected in the inactivated vaccine group at day 7, and at 14 dpi, neutralizing antibody levels were significantly lower than those in all attenuated vaccine groups. At 21 dpi, no significant differences were observed among the groups. At 28 dpi, neutralizing antibody levels in the inactivated vaccine group were extremely significantly higher than those in all other attenuated vaccine groups, likely due to the rapid increase induced by the booster immunization administered at 21 dpi. At 35 dpi, no significant differences were observed among the groups. At 42 dpi, neutralizing antibody levels in the ∆T3 high-dose group were significantly higher than those in the ∆T3 low-dose group. At 49 dpi, neutralizing antibody levels in the inactivated vaccine group began to decline, whereas no such decline was observed in the attenuated vaccine groups.

Taken together, these results indicate that prior to the booster immunization of the inactivated vaccine (before 21 dpi), neutralizing antibody levels in the inactivated vaccine group increased more slowly than those in the gene-deleted vaccine groups. However, after booster immunization, neutralizing antibody levels in the inactivated vaccine group surpassed those in the attenuated vaccine groups. Following challenge, the gene-deleted vaccines elicited a rapid rise in neutralizing antibodies, reaching levels comparable to those induced by the inactivated vaccine. The duration of neutralizing antibody maintenance was relatively shorter for the inactivated vaccine compared with the attenuated vaccines.

#### 3.4.2. Kinetics of Specific Antibody Responses

The gB-specific antibody response was assessed using a commercial blocking ELISA kit (Figure 5A). Prior to immunization (day 0), all groups were seronegative for BoHV-1 *gB* antibodies. Significant differences were observed only within the first three weeks post-immunization (Figure S1), and from the fourth week onward, no significant differences were found among the groups. Following immunization, all gene-deleted strain groups seroconverted to gB antibody-positive by day 7 post-immunization. At this time point, both the high- and low-dose gene-deleted strain groups exhibited significantly higher blocking rates than the inactivated vaccine group. On day 14, both ∆T3-immunized groups and ∆g3-immunized groups showed significantly higher blocking rates compared with the inactivated vaccine group. By day 21, the ∆T3 high dose group and both ∆g3 groups display significantly higher blocking rates than the inactivated vaccine group, respectively. From day 28 onward, no significant intergroup differences were detected, indicating comparable antibody persistence across all vaccine groups. These results demonstrate that, compared with the commercial inactivated vaccine, the gene-deleted strains elicited a more rapid *gB*-specific antibody response during the early phase post-immunization. In contrast, the inactivated vaccine required a booster to achieve antibody levels comparable to those of the gene-deleted strains. Additionally, the titers of gB-specific antibodies were measured in each group on day 28 post-immunization, and the results are shown in Figure 5B. The commercial inactivated vaccine group exhibited the highest antibody titers, reaching approximately 1:1024. The ∆T3 high-dose group (1.0 × 10^7^ TCID_50_) and both the ∆g3 groups (1.0 × 10^7^ TCID_50_ and 1.0 × 10^6^ TCID_50_) showed intermediate titers, ranging between 1:512 and 1:1024. The ∆T3 low-dose group (1.0 × 10^6^ TCID_50_) had the lowest antibody titers, at approximately 1:128, which were significantly lower than those of the other four immunized groups.

In this study, we also measured the gE antibody levels in rabbits from each group after immunization and challenge using a commercial gE blocking ELISA kit. The results further confirm the deletion of the *gE/gI* genes in the constructed attenuated vaccine strains. As shown in Figure 5D, all groups immunized with attenuated vaccines tested negative for *gE* antibodies prior to challenge (28 dpi), with blocking rates below 55%, indicating that no antibodies against the gE protein were produced in the immunized animals. This finding is consistent with the successful deletion of the *gE/gI* genes in the vaccine strains. In contrast, *gE* antibody positivity began to appear in the attenuated vaccine groups in the second week post-challenge, and antibody levels gradually increased over time. This phenomenon can be attributed to the challenge virus (BoHV-1 BC01), which carries an intact gE gene and induces the production of specific antibodies against the *gE* protein during infection, leading to seroconversion after challenge.

Notably, the *gE* antibody response in the inactivated vaccine group followed a trend similar to that of *gB* antibodies: positivity appeared in the third week after primary immunization, and *gE* antibody levels increased rapidly after booster immunization (21 dpi), subsequently remaining stable. This result is consistent with expectations, as the inactivated vaccine is prepared from complete virus particles containing the *gE* protein, which can induce *gE* antibody production in vaccinated animals. This further confirms the limitation of inactivated vaccines in implementing the DIVA (Differentiating Infected from Vaccinated Animals) strategy.

#### 3.4.3. Clinical Observations and Viral Shedding Following Challenge

**Figure 6:**
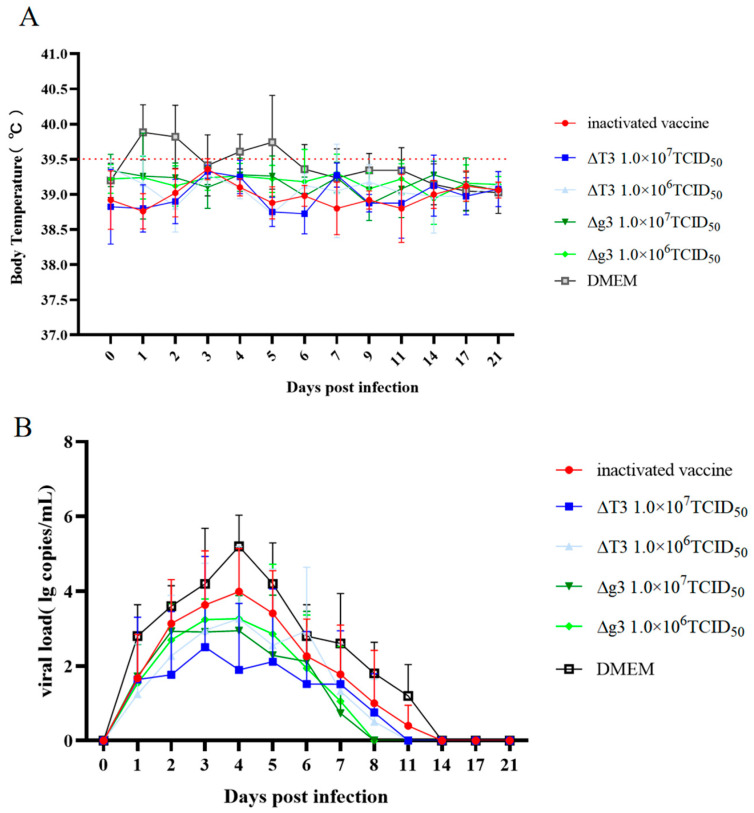
Post-challenge rectal temperature. (**A**) Post-challenge rectal temperature changes in rabbits infected with the BoHV-1 BC01 strain. (**B**) Post-challenge viral load (BoHV-1 BC01) in rabbit nasal swabs. The red line indicates the threshold between normal rectal temperature and the febrile state.

At 28 days post-immunization, all rabbits were challenged with the same dose (1.0 × 10^8^ TCID_50_) of the BoHV-1 BC01 strain. Morbidity and mortality were monitored over a 3-week period. Body temperatures in all immunized groups remained within the normal range without any febrile response (Figure 6A). In the commercial inactivated vaccine group, one rabbit exhibited clinical symptoms lasting for 3 days. No clinical symptoms were observed in the ∆T3 high-dose group (1.0 × 10^7^ TCID_50_). In the ∆T3 low-dose group (1.0 × 10^6^ TCID_50_), one rabbit showed symptoms that persisted for 6 days. Both the ∆g3 high-dose (1.0 × 10^7^ TCID_50_) and low-dose (1.0 × 10^6^ TCID_50_) groups remained asymptomatic. In contrast, all five rabbits in the non-immunized challenge control group exhibited clinical signs accompanied by elevated body temperature, with symptoms lasting approximately 9 days (Table 6). Based on the assessment of body temperature changes and clinical symptoms, all five vaccine groups conferred immune protection. Among them, the ∆g3 high-dose group, ∆g3 low-dose group, and ∆T3 high-dose group demonstrated superior protective efficacy, while the ∆T3 low-dose group and the commercial inactivated vaccine group showed relatively poorer protection.

Following challenge, nasal swabs were collected from rabbits in each group. Viral nucleic acids were extracted and detected by quantitative real-time PCR (qPCR). The results are presented in Figure 6B. In the commercial inactivated vaccine group, the viral load peaked at approximately 10^3.9^ copies/mL on 4 dpi, gradually decreased thereafter, and became undetectable by 11 dpi. In the ∆T3 high-dose group (1.0 × 10^7^ TCID_50_), the viral load peaked at approximately 10^2.5^ copies/mL on 3 dpi and shedding ceased by 8 dpi. In the ∆T3 low-dose group (1.0 × 10^6^ TCID_50_), the viral load peaked at approximately 10^3.3^ copies/mL on 4 dpi, and shedding ended by 8 dpi. In the ∆g3 high-dose group (1.0 × 10^7^ TCID_50_), the viral load peaked at approximately 10^2.9^ copies/mL on day 3 and shedding ceased by day 7. In the ∆g3 low-dose group (1.0 × 10^6^ TCID_50_), the viral load peaked at approximately 10^3.0^ copies/mL on 3 dpi, and shedding ended by 7 dpi. In the non-immunized challenge control group, viral shedding was detected beginning on 1 dpi, peaked at approximately 10^5.2^ copies/mL on 4 dpi, and became undetectable by 11 dpi. Although viral shedding was observed in all immunized groups, the viral loads were significantly lower (a decrease of approximately 100-fold) and the shedding durations were notably shorter (3–4 days) compared with those in the non-immunized control group. Based on the viral shedding data, the ∆T3 high-dose group exhibited the best protective efficacy, followed in descending order by the ∆g3 high-dose group, the ∆T3 low-dose group, the ∆g3 low-dose group, and the commercial inactivated vaccine group.

#### 3.4.4. Lung Histopathology

**Figure 7:**
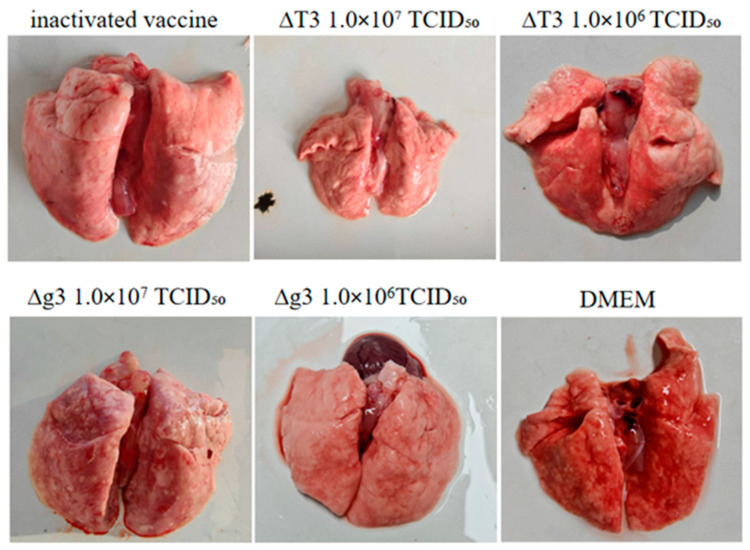
Gross pathology of rabbit lungs at necropsy.

**Figure 8:**
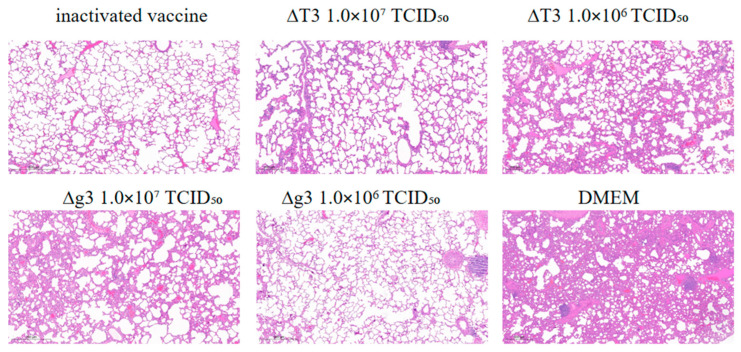
Histopathological images of lung tissues after BoHV-1 BC01 challenge stained by H&E. The scale size is 500 μm.

Macroscopic lung lesions were scored as described in Section 2.8, and representative images from each group are shown in Figure 7. Histopathological changes in lung tissues were evaluated using a 0–4 scoring system, based on alveolar wall thickening, architectural disruption, capillary congestion, inflammatory infiltration, alveolar dilation, and intra-alveolar exudate. As shown in Figure 8, the non-immunized challenge control group showed the most severe lesions (mean score = 3.4). The ∆T3 low-dose group exhibited mild but notable changes, including mild alveolar wall thickening, disrupted architecture, capillary congestion, sparse inflammatory cell infiltration, and mild alveolar dilation (mean score = 2.0). In contrast, the ∆T3 high-dose, ∆g3 high-dose, ∆g3 low-dose, and inactivated vaccine groups all displayed relatively milder histopathological alterations, with continuous, thin alveolar walls, narrow interstitium, normal alveolar epithelial cells, and no edema or inflammatory exudate. Their mean scores were as follows: ∆g3 high-dose (0.5), ∆T3 high-dose (0.6), ∆g3 low-dose (0.6), and inactivated vaccine (0.7).

## 4. Discussion

Infectious bovine rhinotracheitis (IBR), first reported in Europe in the early 20th century, has since spread globally, with some countries, such as Austria, Denmark, Finland, Sweden, Switzerland, and Norway, successfully eradicating the disease. Meanwhile, countries including Australia, Belgium, Canada, India, Poland, Turkey, and the United States have implemented vaccination-based control strategies [18,19]. In China, recent serological surveys and meta-analyses estimate the seroprevalence of BoHV-1 at the herd level, ranging from 24% to 68%, with a pooled estimate of approximately 40% [20]. Vaccination remains the most effective strategy for controlling BoHV-1-related diseases. Inactivated vaccines, which offer a high safety profile and are safe for use in pregnant cows, generally require two doses to induce protective immunity [21,22]. Although inactivated vaccines elicit robust humoral immune responses, they fail to induce strong cellular immunity. Furthermore, the duration of immunity provided by inactivated vaccines is typically shorter than that conferred by modified-live virus (MLV) vaccines. Inactivated vaccines administered via intramuscular or subcutaneous routes do not induce mucosal immunity [16]. In contrast, MLV vaccines allow differentiation between infected and vaccinated animals (DIVA principle). Considering these aspects, this study aimed to develop two gene-deleted vaccine candidates to provide references for the prevention and control of IBR.

Although cattle are the natural host of BoHV-1 and represent the ideal animal model for vaccine evaluation, their use is associated with several limitations. These include a long experimental timeline, high costs, complex handling procedures, and susceptibility to environmental and experimental variability, which introduce numerous uncontrollable factors and subjective bias in certain endpoint assessments. More critically, the high seroprevalence of IBR makes it extremely difficult to screen for seronegative cattle, thereby greatly restricting the feasibility of cattle-based trials. In contrast, the rabbit model is the most well-established experimental animal model for BoHV-1 research. Rock et al. (1982) [23] developed a rabbit model via conjunctival inoculation with BoHV-1 and demonstrated that all inoculated rabbits established latent infection, with spontaneous viral reactivation observed. This model has since been widely used to investigate the mechanisms of latency and reactivation [23]. More recently, using the rabbit model, a bivalent vaccine combining an attenuated Mycoplasma bovis HB150 strain and a BoHV-1 *gG*^−^/*tk*^−^ vaccine strain demonstrated safety and protective efficacy, thereby providing a valuable experimental platform for subsequent application in cattle [24]. It should be noted that rabbits are not the natural host of BoHV-1, and viral replication in rabbits is significantly restricted compared with cattle. The semi-permissive nature of the rabbit model explains why the viral loads observed in the non-immunized control group are lower than those typically seen in cattle, which is consistent with other rabbit model studies [25,26].

In this study, two triple-gene deletion BoHV-1 strains, ∆*TK*-∆*gE/gI* (∆T3) and ∆*gG*-∆*gE/gI* (∆g3), were successfully constructed and rescued. Through systematic in vitro characterization and evaluation in a rabbit model, we preliminarily demonstrated that both candidate vaccines possess favorable safety and immunogenicity profiles, providing promising candidate strains for the development of novel IBR marker vaccines (DIVA vaccines). In vitro results demonstrate that both deletion mutants exhibited growth kinetics similar to the wild-type strain, while plaque areas were markedly diminished. This confirms the critical role of the *gE/gI* complex in cell-to-cell viral spread [12]; its deletion effectively restricts the lateral spread of the virus within infectious foci, consistent with findings in other alphaherpesviruses such as pseudorabies virus and equine herpesvirus [27,28,29,30,31]. Reduced plaque size serves as a typical in vitro indicator of attenuated virulence, providing a theoretical basis for subsequent in vivo safety results.

The rabbit safety evaluation revealed two key trends. First, the triple-gene deletion mutants exhibited significantly improved safety profiles compared with the single-gene deletion parental strains. While the single-gene deletion mutants (∆*TK*, ∆*gG*, ∆*gE/gI*) still induced febrile responses and viral shedding to some extent, the triple-gene deletion mutants, especially ∆T3 and the low-dose ∆g3 group, exhibited no clinical symptoms, with extremely low shedding titers and short durations. This highlights the additive attenuating effect of multi-gene deletion. Similar findings have also been reported by Balan et al. in studies on single-gene mutants of herpes simplex virus and by Silva et al. in studies on single-gene deletion mutants of BoHV-5 [12,32]. Second, ∆T3 showed a superior safety profile compared with ∆g3. While the high-dose ∆g3 group exhibited suboptimal safety, the low-dose group performed comparably to ∆T3 in terms of safety. This difference likely arises from the unique role of the *TK* gene in the alphaherpesvirus life cycle. *TK* plays a key role in establishing latent infection and reactivation in neuronal ganglia [15]; its deletion is believed to attenuate viral neurotropism and pathogenic potential. In contrast, *gG*, a secreted glycoprotein, primarily influences viral cell-to-cell spread efficiency and immunomodulation. Its deletion may not restrict viral replication at the infection site as profoundly as *TK* deletion. Thus, the deletion of *TK* likely confers a higher safety margin for the vaccine.

In terms of immunogenicity, both live attenuated vaccines provided substantial protection against virulent challenge, as evidenced by mild or absent clinical symptoms post-challenge, normal body temperatures, a more than 100-fold reduction in viral shedding, a significantly shortened shedding period (approximately 3 days), and attenuated pathological damage in the lungs and trachea. These indicators are consistent with the multi-parameter evaluation system for vaccines established by the World Organisation for Animal Health (OIE) [33]. In terms of neutralizing antibodies, the gene-deleted vaccines induced higher levels during the early stage of immunization and elicited a rapid increase post-challenge, with longer persistence, demonstrating the strong capacity of gene-deleted vaccines to generate immune memory and anamnestic responses. Regarding specific antibody levels, the gene-deleted vaccines induced more rapid increases in gB antibody levels, with gB antibody positivity detectable by day 7 post-immunization, whereas the inactivated vaccine did not test positive until day 14 post-immunization. These results confirm that live attenuated vaccines, by virtue of their limited replication capacity, more effectively mimic natural infection, thereby eliciting more balanced humoral immunity, which is crucial for clearing intracellular virus and providing long-term protection.

Double-gene deletion vaccines (mainly *gG*/*TK* or *gE*/*TK*) are widely studied. They show favorable safety and robust immunogenicity. After challenge, vaccinated animals exhibit reduced viral shedding, milder pulmonary lesions, and superior protection compared with inactivated vaccines [34,35]. Building upon these findings, the present study constructed two triple-gene deletion mutants (∆T3 and ∆g3) to further enhance vaccine safety and marker-based differentiation capacity through synergistic multi-gene deletion. Rabbit model evaluations demonstrated that these triple-gene deletion mutants retained robust immunogenicity while exhibiting improved safety profiles compared with single-gene deletion mutants. Among them, the ∆T3 high-dose and ∆g3 low-dose regimens were identified as the most promising vaccine candidates. A limitation of this study is the lack of direct cellular immune data (e.g., IFN-γ and TNF-α levels) due to technical difficulties with the assays. Future studies should include proper cellular immunity evaluation using validated kits and fresh or carefully handled samples to further support the superior immunogenicity of the gene-deletion vaccines, as well as validation of these candidates in cattle and the development of companion diagnostic assays to facilitate their progression toward practical vaccine applications.

## 5. Conclusions

In conclusion, the two triple-gene deletion recombinant viruses (∆T3 and ∆g3) developed in this study exhibit excellent safety and immunogenicity in the rabbit model, supporting their further evaluation as promising vaccine candidates against infectious bovine rhinotracheitis. Furthermore, with favorable biological characteristics and genetic stability, they are suitable for large-scale production and commercialization. Further studies in target animals and clinical trials will promote their application as practical vaccines against infectious bovine rhinotracheitis.

## Figures and Tables

**Table 1:** Main reagents and consumables used in this study.

Reagent	Manufacturer
L-(+)-Arabinose (A61007-0100)	Wuhan Puno Sai Biotechnology Co., Ltd. (Wuhan, China)
DMEM medium	Wuhan Puno Sai Biotechnology Co., Ltd. (Wuhan, China)
ProFection Mammalian Transfection System (E1200)	Promega Corporation (Madison, WI, USA)
Plasmid Mini Kit (D6943-02)	Omega Bio-tek, Inc. (Norcross, GA, USA)
MiniBEST DNA Fragment Purification Kit (9761)	TAKARA Bio Inc. (Kusatsu, Japan)
MiniBEST Agarose Gel DNA Extraction Kit (9762)	TAKARA Bio Inc. (Kusatsu, Japan)
MiniBEST Viral RNA/DNA Extraction Kit (9766)	TAKARA Bio Inc. (Kusatsu, Japan)
Low-melting-point agarose (A104063)	Shanghai Aladdin Biochemical Technology Co., Ltd. (Shanghai, China)
2 × TransFast Taq PCR SuperMix (S102)	Beijing TransGen Biotech Co., Ltd. (Beijing, China)
2 × TOROBlue Flash KOD Dye Mix	Toroivd Technology Co., Ltd. (Shanghai, China)

**Table 2:** Main instruments used in this study.

Instrument	Manufacturer
Optical microscope (E200)	Nikon Corporation (Tokyo, Japan)
Fluorescence inverted microscope (TE2000-U)	Nikon Corporation (Tokyo, Japan)
Gene Pulser Xcell electroporation system	Bio-Rad Laboratories, Inc. (Hercules, CA, USA)
CO_2_ incubator (311)	Thermo Fisher Scientific Inc. (Waltham, MA, USA)
Automatic nucleic acid extractor	Jifan Biotechnology (Changzhou) Co., Ltd. (Changzhou, China)
Gel imaging system (GelDoc XR+)	Bio-Rad Laboratories, Inc. (Hercules, CA, USA)
Electronic balance (XPR56DR/AC)	Shanghai Precision Scientific Instrument Co., Ltd. (Shanghai, China)
Thermostatic shaking metal bath	Bioer Technology Co., Ltd. (Hangzhou, China)

**Table 3:** Experimental Protocol for Safety Evaluation in Rabbits.

Group	Number	Immunogen	Dose (TCID_50_/mL)	Route of Inoculation
1	5	∆T3	1.0 × 10^7^	intramuscular injection
2	5	∆T3	1.0 × 10^6^	intramuscular injection
3	5	∆g3	1.0 × 10^7^	intramuscular injection
4	5	∆g3	1.0 × 10^6^	intramuscular injection
5	5	rBoHV-1-∆gG	1.0 × 10^6^	intramuscular injection
6	5	rBoHV-1-∆TK	1.0 × 10^6^	intramuscular injection
7	5	rBoHV-1-∆gE/gI	1.0 × 10^6^	intramuscular injection
8	5	DMEM	1 mL	intramuscular injection
9	5	wt BoHV-1	1.0 × 10^6^	intramuscular injection
10	5	Inactivated vaccine	according to the instructions	intramuscular injection

**Table 4:** Immune Protection Evaluation Protocol.

Group	Number	Challenge Strain	Dose (TCID_50_/mL)	Challenge Route
∆T3 1.0 × 10^7^	5	BoHV-1 BC01	1.0 × 10^8^	intranasal (i.n.)
∆T3 1.0 × 10^6^	5	BoHV-1 BC01	1.0 × 10^8^	intranasal (i.n.)
∆g3 1.0 × 10^7^	5	BoHV-1 BC01	1.0 × 10^8^	intranasal (i.n.)
∆g3 1.0 × 10^6^	5	BoHV-1 BC01	1.0 × 10^8^	intranasal (i.n.)
inactivated vaccine	5	BoHV-1 BC01	1.0 × 10^8^	intranasal (i.n.)
DMEM	5	BoHV-1 BC01	1.0 × 10^8^	intranasal (i.n.)

**Table 5:** Clinical symptoms observed in rabbits infected with different strains.

Group	Group Number									
	Onset of Symptoms (dpi)					Symptom Duration (dpi)				
	1	2	3	4	5	1	2	3	4	5
∆T3 1.0 × 10^7^ TCID_50_	0	0	0	0	0	0	0	0	0	0
∆T3 1.0 × 10^6^ TCID_50_	0	0	0	0	0	0	0	0	0	0
∆g3 1.0 × 10^7^ TCID_50_	0	2	3	0	0	0	5	6	0	0
∆g3 1.0 × 10^6^ TCID_50_	0	0	1	0	0	0	0	3	0	0
rBoHV-1-∆TK	0	2	0	0	1	0	6	0	0	5
rBoHV-1-∆gG	2	3	2	0	1	7	6	6	0	5
rBoHV-1-∆gE/gI	1	0	0	2	2	6	0	0	4	5
WT BoHV-1	1	2	2	1	3	8	10	11	9	10

**Table 6:** Clinical symptoms observed in each group post-challenge.

Group	Inactivated Vaccine					∆T3 1.0 × 10^7^ TCID_50_					∆T3 1.0 × 10^6^ TCID_50_					∆g3 1.0 × 10^7^ TCID_50_					∆g3 1.0 × 10^6^ TCID_50_					DMEM				
Group number	1	2	3	4	5	1	2	3	4	5	1	2	3	4	5	1	2	3	4	5	1	2	3	4	5	1	2	3	4	5
Onset of symptoms	2	0	0	0	0	0	0	0	0	0	0	0	2	0	0	0	0	0	0	0	0	0	0	0	0	2	1	2	1	3
Symptom duration	3	0	0	0	0	0	0	0	0	0	0	0	6	0	0	0	0	0	0	0	0	0	0	0	0	9	9	8	10	11

## Data Availability

The data supporting the findings of this study are available within the article. Further inquiries can be directed to the corresponding authors.

## Supplementary Materials

The following supporting information can be downloaded at: https://www.mdpi.com/article/10.3390/ani16111606/s1. Table S1: Primers used in this study. Figure S1: Comparison of gB antibody blocking rates among different immunization groups during the first three weeks post-immunization. (A) Day 7; (B) Day 14; (C) Day 21. Significance levels are indicated as * *p* < 0.05, ** *p* < 0.01, and *** *p* < 0.001.
