# Supplementary material for: Development and Immune Efficacy Evaluation of Two Live Triple-Gene-Deleted Vaccine Candidates Against Bovine Herpesvirus Type 1
Source: Animals (Basel). 2026 May 25;16(11):1606. doi: 10.3390/ani16111606 (PMC13255727; doi:10.3390/ani16111606)
Supplement: Supplementary file 1 [file animals-16-01606-s001.zip › animals-4251919-supplementary.pdf]

Table S1. Primers used in this study

Primers	Sequence(5’-3’)	Product
TK-ΔF	CGGATCCCGGCGCGCAGGCGCGCACGTCGGTCGCGGTCGCGCGCCAGCT GCCCCTGCGCTCGCTCggatgacgacgataagtaggga	ΔTK targeting fragment
TK-ΔR	CGATCGGGGCGCAAATGCAGCGAGCGAGCGAGCGCAGGGGCAGCTGGC GCGCGACCGCGACCGACgggtaatgccagtgttacaacc	
gI/gE-ΔF	GCGACTCAAGCCATTGCCGCGACCTTGTCCTCCGGCGCGCTCGCGGCGCC CCCCCCCCCGCGCGCggatgacgacgataagtaggga	ΔgI/gE targeting fragment
gI/gE-ΔR	CGGGTGCTTTCCGTCAGACGGCACAGCGCGCGGGGGGGGGGCGCCGC GAGCGCGCCGGAGGACAgggtaatgccagtgttacaacc	
gG-ΔF	AACGCGAGCGAACGCGAGCGCAAGCGCGAGCACACGACTGCGATCTCG CCGGCACCCCACGCCGCggatgacgacgataagtaggga	ΔgG targeting fragment
gG-ΔR	GTAAACGCGGGACAGCGGGGTCGGGGCGGCGTGGGGTGCCGGCGAGAT CGCAGTCGTGTGCTCGCgggtaatgccagtgttacaacc	
TK-JD-F	TTCATGTTCGTTGAAAAACGGCACGTCTTCAG	TK deletion identified
TK-JD-R	CATTTCCCACTCTTCGATTTCGTATCGGGCGT	
gG-JD-F	CCGGGGGTTTCCCGCAAAACTG	gG deletion identified
gG-JD-F	AATGTCGGCCCTTGCATGTTC	
gI/gE-JD-F	CAGAAAGCCAAAAAAGCTGC	gI/gE deletion identified
BoHV-1/UL1/F	CGAAGGCGCTATTGAGGACT	
BoHV-1/UL1/R	CGCTGAAGATATAAGGCGGGT	
BoHV-1/UL1/P	TCTCGATACACTGGAGTGTCGGCAAGC	

Lowercase letters indicate the amplification primers used to introduce the Kan sequence from the pEP-Kan-S plasmid. The uppercase portions represent sequences homologous to the regions flanking the gene to be knocked out.

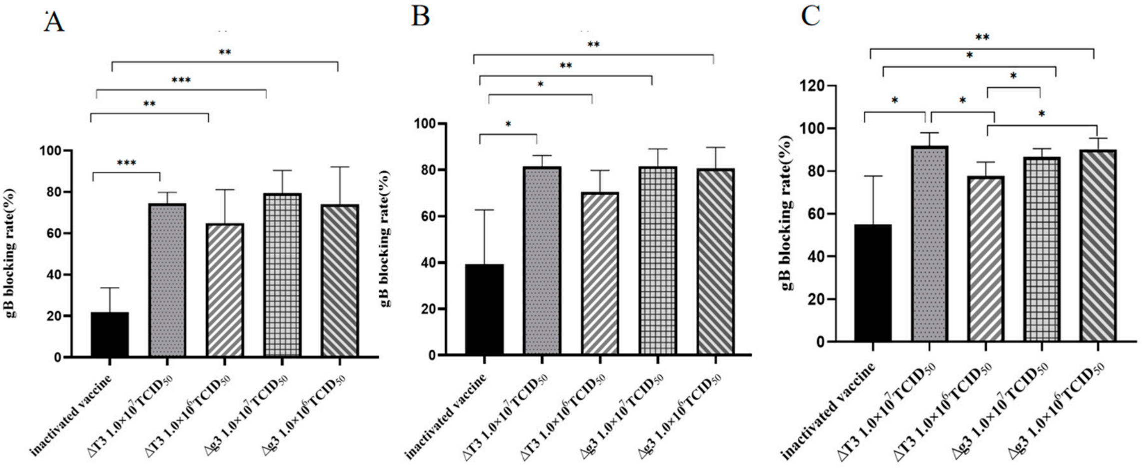

Figure S1. Comparison of gB antibody blocking rates among different immunization groups during the first three weeks post-immunization. (A) Day 7; (B) Day 14; (C) Day 21. Significance levels are indicated as * $p < 0.05$, ** $p < 0.01$, and *** $p < 0.001$.
